# Interaction of *Garcinia cambogia* (Gaertn.) Desr. and Drugs as a Possible Mechanism of Liver Injury: The Case of Montelukast

**DOI:** 10.3390/antiox12091771

**Published:** 2023-09-16

**Authors:** Silvia Di Giacomo, Antonella Di Sotto, Ester Percaccio, Erica Scuotto, Cecilia Battistelli, Gabriela Mazzanti, Francesca Menniti-Ippolito, Ilaria Ippoliti

**Affiliations:** 1 Department of Food Safety, Nutrition and Veterinary Public Health, National Institute of Health, Viale Regina Elena 299, 00161 Rome, Italy; 2 Department of Physiology and Pharmacology “V. Erspamer”, Sapienza University of Rome, P.le Aldo Moro 5, 00185 Rome, Italy; ester.percaccio@uniroma1.it (E.P.); scuotto.1635570@studenti.uniroma1.it (E.S.); gabriela.mazzanti@uniroma1.it (G.M.); 3 Department of Molecular Medicine, Sapienza University of Rome, Viale Regina Elena 324, 00161 Rome, Italy; cecilia.battistelli@uniroma1.it; 4 National Centre for Drug Research and Evaluation, National Institute of Health, Viale Regina Elena 299, 00161 Rome, Italy; francesca.menniti@iss.it (F.M.-I.); ilaria.ippoliti@iss.it (I.I.)

**Keywords:** *Garcinia cambogia*, herb-induced liver injury, phytovigilance, herb-drug interactions, synergism, oxidative stress, dietary supplements

## Abstract

Overweight and obesity prevalence has increased worldwide. Apart from conventional approaches, people also resort to botanical supplements for reducing body weight, although several adverse events have been associated with these products. In this context, the present study aimed at evaluating the toxicity of *Garcinia cambogia*-based products and shedding light on the mechanisms involved. The suspected hepatotoxic reactions related to *G. cambogia*-containing products collected within the Italian Phytovigilance System (IPS) were examined. Then, an in vitro study was performed to evaluate the possible mechanisms responsible for the liver toxicity, focusing on the modulation of oxidative stress and Nrf2 expression. From March 2002 to March 2022, the IPS collected eight reports of hepatic adverse reactions related to *G. cambogia*, which exclusively involved women and were mostly severe. The causality assessment was probable in three cases, while it was possible in five. In the in vitro experiments, a low cytotoxicity of *G. cambogia* was observed. However, its combination with montelukast greatly reduced cell viability, increased the intracellular ROS levels, and affected the cytoplasmic Nrf2 expression, thus suggesting an impairment of the antioxidant and cytoprotective defenses. Overall, our results support the safety concerns about *G. cambogia*-containing supplements and shed light on the possible mechanisms underpinning its hepatotoxicity.

## 1. Introduction

The liver represents a vital organ in the human body, playing a crucial role in several physiological functions, including the synthesis and storage of nutrients, metabolism, and detoxification of harmful substances [1,2]. Therefore, injuries at the liver level greatly impair global wellness, leading to severe reactions and sometimes to death [1]. Regardless of etiology, oxidative stress seems to be the most important pathogenic event in liver diseases [3]. 

Reactive oxygen species (ROS) are physiologically produced in the human body as a byproduct of mitochondrial respiration and are exploited by cells to exert biological properties, such as the regulation of specific genes’ expression or as effector molecules against pathogens [4,5,6]. Low to moderate levels of ROS in the cells are ensured by the endogenous antioxidant systems. However, the impairment of defense systems can lead to the accumulation of ROS, which can substantially damage cell structure and functions, including lipids, DNA, proteins, and related signaling pathways, leading to apoptosis or necrosis of hepatic cells [4,7]. In particular, the reaction of ROS with lipids determines the production of reactive intermediates, namely malondialdehyde and 4-hydroxynonenal, which form covalent adducts with proteins, DNA, and phospholipids, thus resulting in cell death. Moreover, these species may alter different signaling pathways involved in cell proliferation, differentiation, and apoptosis (e.g., extracellular signal-regulated kinase1/2, c-Jun N terminal kinase, p38 mitogen-activated protein kinase) by targeting protein thiols, thus modifying their structure and function [7]. In addition, oxidative stress can induce endoplasmic reticulum (ER) stress, iron-overload-mediated ferroptosis, and pyroptosis, and activate the NLRP3 inflammasome [8]. In this regard, under severe oxidative stress, ROS induce the overexpression of proinflammatory genes with the consequent release of chemical mediators, namely eicosanoids, cytokines, chemokines, and nitric oxide, that induce tissue damage and augment oxidative stress. This results in a vicious cycle, where increased oxidative stress and inflammatory lesions promote the pathogenesis of liver diseases [7].

As well as hepatocytes (70% of the liver cell population), other hepatic non-parenchymal cells, namely intrahepatic cholangiocytes, may contribute to the pathogenesis of liver injury [9]. Indeed, these cells are involved in bile secretion and liver regeneration; moreover, they protect the liver from the accumulation of toxicants through the biliary defense [10]. Therefore, oxidative damage at the cholangiocytes level could trigger or exacerbate liver diseases. Notably, bile duct injury has become one of the leading causes of acute liver failure [10]. 

Several risk factors, including obesity, viruses, alcohol, drugs, and also botanicals, have been highlighted as inducers of hepatic diseases [7,11]. In particular, liver damage due to the consumption of botanicals, also known as herb-induced liver injury (HILI), has greatly increased in the last few years accounting for about 25% of the total incidence of liver injury, thus raising public concerns about their safety [12,13]. Moreover, gender differences have been highlighted, with the occurrence of HILI being higher in females (32.7%) with respect to males (20.6%). In addition, it has been estimated that about 7.7% of hepatotoxicity cases are ascribable to the concomitant intake of conventional drugs and botanicals [13]. China represents the nation with the highest proportion of HILI (35.7%), followed by South Korea (24.4%), Western nations (8.1%), and Southeast Asia (5.4%) [13]. Common examples of botanicals causing HILI are *Tripterygium wilfordii* Hook F. [10], *Polygonum multiflorum* [PM] Thunb. [14], Green tea [15], and *Curcuma longa* L. [16]. Recently, *Garcinia cambogia* (Gaertn.) Desr. (syn. *G. gummi-gutta*)-containing supplements have also raised some concerns [17,18,19,20,21,22,23]. 

*G. cambogia* (Clusiaceae family), also known as Malabar tamarind, is a plant native to India and Southeast Asia [17], used traditionally as a remedy to treat gastrointestinal problems, diarrhea, and ulcers [24]. It is endowed with several beneficial properties, including anti-ulcerogenic, antioxidant, antidiabetic, antiinflammatory, and anticancer ones [24]. Nowadays, extracts from its immature fruit are mainly used in dietary supplements for weight loss or weight maintenance. Various biological active compounds have been found in *G. cambogia*, among which garcinol, isogarcin, mangostin, and xanthoquimol; in particular, (−)-hydroxycitric acid (HCA), mainly present in the fruit rind (20–30% of the dry weight) [25], is the most investigated, being considered responsible for the slimming properties [17]. Indeed, it has been highlighted that HCA is able to reduce food intake by regulating the serotonin level and to promote the energy expenditure by inducing metabolic modifications (i.e., fat oxidation increase, de novo lipogenesis decrease, and hepatic glycogenesis stimulation). Moreover, HCA inhibits the adenosine triphosphate (ATP)-citrate lyase enzyme, thus reducing the synthesis of fatty acids [17]. 

The clinical efficacy of *G. cambogia* in weight management has been investigated by several studies, albeit obtaining conflicting results [17]. However, supplementation with *G. cambogia* was concluded to be safe since only minor side effects were registered (e.g., leg cramps, heartburn, diarrhea, headaches, general weakness). Despite this, a growing number of hepatotoxicity events have been reported in the literature, sometimes serious enough to require liver transplantation [17]. Among the most recent, the case described by Yousaf et al. [18] is noteworthy, in which acute liver failure occurred in a 21-year-old female with noted obesity (body mass index 40.34 kg/m^2^) after the consumption of *G. cambogia* (1400 mg/day) for 4 weeks. The causality assessment was certain [18]. Another hepatotoxicity report related to the consumption of *G. cambogia* (daily dose ranging from 1000 to 2000 mg) was described by Ordeig et al. [19]. A 64-year-old female obese patient (body mass index 31) consumed the supplement for about 15 days prior to the onset of symptoms. The liver injury was attributed to *G. cambogia* (very probable or certain causality). In addition, Ferreira and coworkers [26] reported the case of a liver transplant in a 26-year-old obese woman (body mass index of 59.8 kg/m^2^) who had been taking *G. cambogia* (1800 mg/day corresponding to 900 mg of HCA) for 7 months. The causality association was judged as probable considering that she also consumed other supplements among which one contained green tea extract, whose hepatotoxicity is known [15]. Finally, in 2023, Flerova et al. [27] reported the case of *G. cambogia*-induced fulminant cholestatic giant cell hepatitis. A 65-year-old woman consumed the supplement for 3 months prior to hospital admission. The causality association was assessed as probable.

In this context, the present study aimed at investigating the risk of liver injury associated with the use of *G. cambogia* supplements based on the analysis of suspected hepatotoxic reactions in humans and toxicological in vitro studies. To this end, firstly, we examined the suspected hepatotoxic reactions related to the use of *G. cambogia*-containing products collected within the database of the Italian Phytovigilance System (IPS). In order to find a causal relationship between the suspected adverse reaction and the product use, an in-depth study of each report, by applying the WHO-UMC causality assessment scale, was carried out [28]. Based on the analysis of the suspected adverse reactions, an in vitro toxicological study was performed, in order to evaluate the possible mechanisms responsible for the liver toxicity associated with *G. cambogia*, especially focusing on the oxidative stress induction and the modulation of the Nrf2 expression, a key factor involved in the activation of antioxidant defenses and cytoprotective factors [29]. H69 intrahepatic cholangiocyte cells, a well-characterized SV40-transformed human bile duct epithelial cell line originally derived from a normal liver harvested for transplantation [30], were exploited as an experimental model. Indeed, it is known that damage to these cells is often involved in the onset of liver damage [31]. Moreover, while the pro-oxidant effect of *G. cambogia* at the hepatic level has been reported [32], it has never been studied at the bile tissue level. Given the evidence that adverse reactions can often occur as a consequence of herb–drug interactions [13], in our assay, we also evaluate the toxicity risk associated with the coadministration of food supplements containing *G. cambogia* and drugs. In particular, based on the analysis of the spontaneous reports of hepatic adverse reactions collected within IPS, the drug montelukast was selected for the combination studies. Montelukast is a leukotriene receptor antagonist exploited to control asthma and allergic rhinitis symptoms [33]. Although generally considered safe, some cases of liver injury have been attributed to it [34,35]. Therefore, combining montelukast and *G. cambogia* represents an appropriate experimental model in order to highlight a possible hepatotoxicity due to herb–drug interaction.

## 2. Materials and Methods

### 2.1. Phytovigilance Methodology

All spontaneous reports of hepatic adverse reactions (ARs) gathered within the IPS, coordinated by National Institute of Health (NIH), were collected. IPS was set up in 2002 to collect spontaneous reports of suspected ARs referred to products of natural origin in order to improve the information about the safety profile of dietary supplements, and galenic or herbal preparations. Within IPS, spontaneous reports of ARs can be reported online (website www.vigierbe.it (accessed on 10 August 2023)) by health professionals, companies, and citizens. For every report, ARs are coded according to the Medical Dictionary of Regulatory Activities (MedDRA), and the composition of the product (ingredients and dosages) is verified through the label notified at the Italian Ministry of Health.

In the present study, we performed an in-depth analysis of ARs related to *Garcinia cambogia*-containing products collected at the IPS from March 2002 to March 2022. All available information on the reports was retrieved and the demographic, clinical, and pharmacological information was collected and analyzed, according to previously published papers [36]. The causality assessment between the product use and AR occurrence (categorized as certain/definite, probable/likely, possible, unlikely, or unassessable/unclassifiable) was evaluated by a multidisciplinary group according to the standardized case causality assessment criteria of the WHO system. 

### 2.2. In Vitro Toxicological Study

#### 2.2.1. Chemicals

If not otherwise specified, all the substances, among which were 3-(4,5 dimethylthiazol-2-yl)-2,5-diphenyl tetrazolium bromide (MTT; CAS number: 298-93-1; purity ≥ 98%), tert-butyl hydroperoxide (tBOOH; 70% wt in H_2_O), 2,7-dichlorofluorescein diacetate (DCFH-DA; CAS number: 4091-99-0; purity ≥ 97%), anti-NF-E2 primary antibody (ABE413), montelukast (MNT; CAS number: 151767-02-1; purity ≤ 100%), and the solvent dimethyl sulfoxide (DMSO; CAS number: 67-68-5; for molecular biology), were purchased from Merck (Darmstadt, Germany). All the materials used for cell cultures, including the RPMI 1640 medium, fetal bovine serum, cofactors, and antibiotics, were provided by Aurogene (Rome, Italy).

#### 2.2.2. Garcinia Cambogia Extract

A dry extract from *G. cambogia* fruit (extraction solvent: water–drug extract ratio 6:1; CAS number 90045-23-1), containing 60% *w*/*w* hydroxycitric acid, produced by Fontana S.r.l. and kindly provided by Federico De Paolis (Farmacia Valle, Rome, Italy), was used. The sample accomplished all the quality requirements for its use in the food field [37], as shown in the technical data sheet provided by the company (see Supplementary Materials).

After preliminary solubility assays, DMSO (100% *v*/*v*) was selected as the best solvent for extract solubilization. The solvent was used up to a maximum 1% *v*/*v* concentration in the final mixture, which did not cause cytotoxic effects and was completely miscible in the cell medium.

#### 2.2.3. Cell Culture

The nonmalignant human intrahepatic cholangiocytes H69 were exploited as a model to investigate the cytotoxicity of both *G. cambogia* extract and montelukast. This cell line was a kind gift from Romina Mancinelli (Department of Anatomical, Histological, Forensic and Orthopedic Sciences, Sapienza University of Rome, Italy). Normal cholangiocytes were grown under standard conditions (37 °C and 5% CO_2_) according to previously published methods [38]. In particular, RPMI-1640 medium supplemented with 10% fetal bovine serum (FBS), 100 U/mL penicillin, 100 µg/mL streptomycin, and 1% L-glutamine was used for H69 cultivation. Every 4 days, cells were subcultured and the growth medium renewed twice a week, as recommended by the supplier. All experiments were performed when the cells reached the logarithmic growth phase.

#### 2.2.4. Cytotoxicity Assay

To perform the experiments, cells were seeded into 96-well microplates (2 × 10^4^ cells/well) and allowed to grow for 24 h; then, progressive dilutions of *G. cambogia* standardized extract (1–500 μg/mL) and the drug assayed in combination were used. The pro-oxidant agent tert-butyl hydroperoxide (tBOOH; 500 μM) was used as a positive control. Cell viability was measured after 24 h and 72 h of incubation with the samples by the 3-[4,5-dimethylthiazol-2-yl]-2,5-diphenyl tetrazolium bromide (MTT) assay, according to previously published methods [38]. The results were expressed as a percentage of the vehicle control. A treatment was considered cytotoxic when the cell viability was less than 70% with respect to the control [39].

#### 2.2.5. Combination Assay

To perform the assay, the cells, grown as previously described [38], were treated with different concentrations of the extract and the selected drug for 24 h; then, the cell viability was measured by the MTT assay [38]. In order to obtain reproducible data, the assay was carried out three times and, in each experiment, each concentration was tested in triplicate.

#### 2.2.6. Analysis of *G.*-*cambogia*–Drug Interaction

The type of interaction was evaluated by the web-free application SynergyFinder Plus (https://tangsoftwarelab.shinyapps.io/synergyfinder/_w_eb6abad8/#!/dashboard (accessed on 10 August 2023)), which allows simultaneous evaluation of synergistic/antagonistic interactions by applying the Highest Single Agent model reference algorithm [40]. Through the analysis of the experimental data obtained in the cytotoxicity assays, the software allows the computation of a synergy score for each combination. In particular, a synergy score value less than −10 indicates antagonism, between −10 and 10 an additive effect, and larger than 10 synergisms.

#### 2.2.7. Determination of Intracellular Levels of Reactive Oxygen Species (ROS)

The ability of the samples to induce the generation of reactive oxygen species (ROS) was measured by the 2,7-dichlorofluorescein diacetate assay (DCFH-DA), according to Di Giacomo et al. [38]. In each experiment, a vehicle control, corresponding to the basal ROS level, and a positive control (tBOOH, 500 μM), corresponding to the highest oxidation, were included too. The fluorescence was measured at an excitation wavelength of 485 nm and emission wavelength of 528 nm by using the Cytation 1 Cell Imaging Multimode Reader (BioTeK^®^ Instruments Inc., Winooski, VT, USA). Fluorescence intensity was determined by the Gen5™ Microplate Reader and Imager Software 3.11 and normalized with respect to cell number. Results were expressed as an oxidation index, calculated as the ratio between the DCF fluorescence of the sample and vehicle control.

#### 2.2.8. Immunofluorescence Analysis of Nrf2

To perform the analysis, 2 × 10^4^ cells were seeded in a 24-well plate and treated with *G. cambogia* standardized extract (1–500 μg/mL) alone or in combination with the selected drug. After 30 min incubation, the cells were fixed in methanol, washed in phosphate-buffered saline + Tween 20 (PBS-T), incubated in 4% bovine serum albumin (BSA), and then stained using the anti-NF-E2 (ABE413, Merck Millipore, Darmstadt, Germany) primary antibody and Hoechst dye (1 μg/mL) for 1 h at room temperature (RT). After washing, a secondary antibody (Alexa Fluor 594-conjugated Chrom Pure Rabbit igG, Jackson Immuno Research Europe Ltd., Ely, UK) was added at RT in a dark room for 1 h. Finally, the cells were analyzed using a Cytation 1 Cell Imaging Multimode Reader (BioTek, AHSI, Milan, Italy) and the fluorescence quantified, as previously reported [41].

### 2.3. Statistical Analysis

The statistical analysis and data representation were performed by using the GraphPad Prism™ (Version 6.00) software (GraphPad Software, Inc., San Diego, CA, USA). Data are displayed as mean ± standard error (SE) of at least three experiments, in which each treatment was tested at least in triplicate. The one-way analysis of variance (one-way ANOVA), followed by Dunnett’s multiple comparison post-test, was used to analyze a statistically significant difference (*p* value < 0.05) among multiple treatments, while the Student’s *t*-test was used to compare the means between two groups.

## 3. Results

### 3.1. Analysis of Spontaneous Reports

From March 2002 to March 2022, 187 reports of hepatic adverse reactions related to the use of natural products were collected by the Italian National Institute of Health within the VigiErbe reporting system, mostly related to food supplements. The reports involved 73 men and 112 women (gender was not specified in 2 cases). The median age was 50 years (range 5–95 years). Overall, 201 hepatotoxic reactions were highlighted, considering that more than one reaction was reported in some cases. In particular, 147 reports (79%) indicated serious liver reactions, which resulted in hospitalizations (n = 127), being life-threatening (n = 13), a serious or permanent disability (n = 1), death (n = 5), or congenital anomaly (n = 1). In the other cases (n = 40), the severity was not indicated. However, the lack of this information could be interpreted as “not serious” adverse reactions considering that in the online form, a specific item about that is not reported.

Within the 187 reports of hepatic adverse reactions, 8 were related to the use of *G. cambogia*-containing food supplements (Table 1) and exclusively involved women with a median age of 46 years (range 39–61 years). All reports were sent by physicians. Serious reactions occurred in seven cases and required hospitalization.

The ARs reported (n = 14) were: massive hepatic necrosis (n = 1), acute hepatitis (n = 5), cholestatic hepatitis (n = 2), abdominal pain (n = 1), hepatomegaly (n = 1), jaundice (n = 1), asthenia (n = 1), nausea (n = 1), and anorexia (n = 1). The duration of use and the latency of the reaction varied from 8 to 522 days (median 1 months) and from 15 to 213 days (median 35 days), respectively. However, in four cases, no information was reported.

Laboratory tests were always performed, showing transaminases values ranging from 67 to more than 3000 U/L, total bilirubin from 0.7 to 30 mg/dL, gamma glutamyl transferase (GGT) from 223 to 341 U/L, and, where indicated, a lack of hepatitis viruses. As for the reason of using the food supplements, in seven cases (88%), weight loss was indicated, while in one, the information was lacking.

Dechallenge was positive in five reports; conversely, and as expected considering the seriousness of the reactions, rechallenge was never reported. Regarding the suspected products, in most of the cases, they were multicomponent (Table 2). Indeed, the number of ingredients ranged from two to eight, and only in one case did *G. cambogia* represent the only component. Moreover, when the information was reported, *G. cambogia* dry extract standardized to contain 60% hydroxycitric acid was used in the supplements (n = 4); although only in two reports was the daily recommended dose indicated. Concomitant diseases were present in five cases. Notably, in one of them, hepatic steatosis, which can predispose to liver failure, was reported.

In four cases, drug intake was also reported; however, it could also be hypothesized in case eight, considering the seriousness of the concomitant pathologies described. The outcomes of the reported ARs were: “complete resolution” (n = 4), “persistent” (n = 1), “recovering” (n = 2), and “death” (n = 1). The causality assessment indicated an association between the product and the reaction, and was assessed as “probable” in three cases (37.5%) and as “possible” in five (62.5%). 

### 3.2. In Vitro Analysis of G. cambogia Hepatotoxicity

Considering the hepatotoxicity reports associated with the consumption of *G. cambogia*-containing products collected within the VigiErbe reporting system, in the subsequent phase of the study, we investigated in vitro the possible mechanisms underlying the observed toxicity. Indeed, although several cases of liver injury associated with *G. cambogia* have been reported in the literature [17,20,22], until now, only one study has investigated the potential underlying mechanisms [32]. Taking into account that in most cases (five out of eight) concomitant drugs were consumed by the subjects, we hypothesized that a herb–drug interaction may contribute to liver damage. Therefore, we focused our attention on the report with the ID number 275, in which the adverse reaction, ended with the patient’s death. The patient took a garcinia-based supplement while she was in therapy with the anti-asthmatic drug montelukast, a drug known to be hepatotoxic. The choice of this case appears useful for toxicological prediction since the supplement was taken in combination with only one drug, unlike other cases in which patients followed a polytherapy. Therefore, we planned to study *G. cambogia* alone and in combination with montelukast.

#### 3.2.1. Cytotoxicity of *G. cambogia* and Montelukast in Human Intrahepatic Cholangiocytes

Preliminary experiments were carried out to evaluate the potential cytotoxicity of *G. cambogia* standardized extract (1–500 μg/mL) and the anti-asthma drug montelukast (0.1–6 μg/mL) in normal H69 cholangiocytes after both 24 h and 72 h of treatment. The concentrations of montelukast to be tested were chosen according to previously published studies [42]. tBOOH was used as a positive control due to its ability to generate oxidative radical species after metabolization by CYP450 [43].

**Figure 1:**
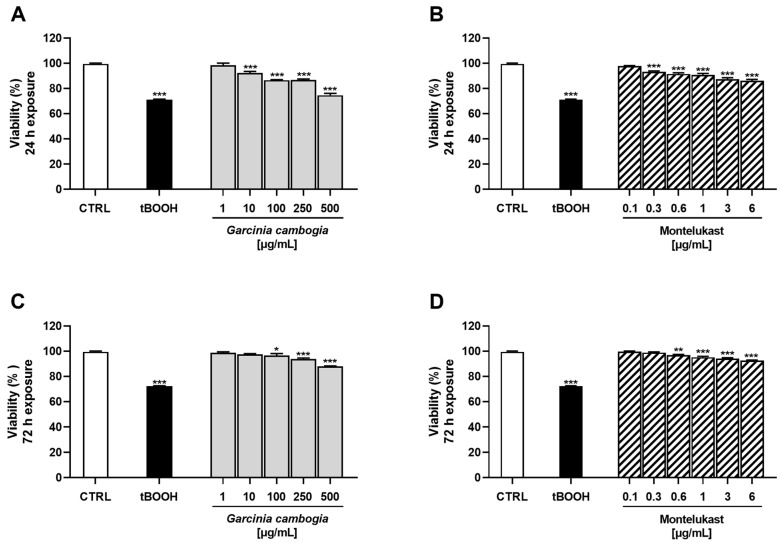
Cytotoxicity of Garcinia cambogia standardized extract (**A**,**C**) and montelukast (**B**,**D**) in human intrahepatic cholangiocytes H69 determined by MTT assay after 24 h and 72 h of exposure. tBOOH: tert-butyl hydroperoxide (500 μM, 3 h of exposure). Values are expressed as mean ± SEM from at least three independent experiments (n = 3). * *p* < 0.05, ** *p* < 0.01, and *** *p* < 0.001 (ANOVA + Dunnett’s multiple comparison post-test), significantly lower with respect to CTRL.

Under our experimental conditions, both *G. cambogia* and montelukast determined a dose-dependent reduction in cell viability, albeit slight, without time-dependence. Indeed, after 24 h of treatment, a maximum cytotoxicity of 25% was observed for *G. cambogia* extract at the concentration of 500 µg/mL, while it was only 14% for montelukast at 6 µg/mL (Figure 1A,B). Conversely, after 72 h of exposure, a lower cytotoxicity was induced by both samples: maximum inhibitions of cell viability of about 12% and 7% were determined by the highest tested concentrations of *G. cambogia* and montelukast, respectively (Figure 1C,D).

As expected, the positive control tBOOH was shown to be the most effective cytotoxic agent by inducing a reduction in cell viability of about 29% at both exposure protocols (Figure 1).

#### 3.2.2. Montelukast Increases *G. cambogia* Cytotoxicity on Human Intrahepatic Cholangiocytes

Based on previous results and considering that in several cases the concomitant consumption of botanicals and conventional drugs determines the triggering of adverse reactions [44], in the subsequent experiments, we investigated the possible cytotoxicity of *G. cambogia* extract when combined with montelukast under 24 h and 72 h exposure protocols.

**Figure 2:**
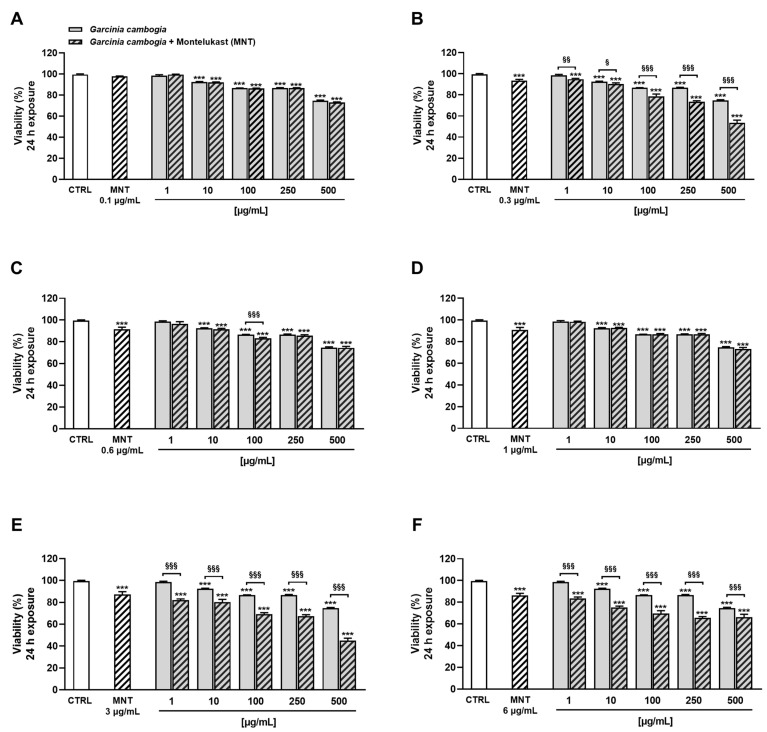
Cytotoxicity of Garcinia cambogia standardized extract in combination with montelukast 0.1 μg/mL (**A**), 0.3 μg/mL (**B**), 0.6 μg/mL (**C**), 1 μg/mL (**D**), 3 μg/mL (**E**), and 6 μg/mL (**F**) in human intrahepatic cholangiocytes H69 determined by MTT assay after 24 h of exposure. Values are expressed as mean ± SEM from at least three independent experiments (n = 3). *** *p* < 0.001 (ANOVA + Dunnett’s multiple comparison post-test), significantly lower with respect to CTRL. ^§^ *p* < 0.05, ^§§^ *p* < 0.01, and ^§§§^ *p* < 0.001 (Student’s *t*-test), significantly lower with respect to the corresponding concentration of Garcinia cambogia standardized extract alone.

Under our experimental conditions, the combined treatment determined a significant reduction in H69 cell viability, although not at all concentrations. In particular, after 24 h of exposure, 0.1 µg/mL and 1 µg/mL montelukast were not able to increase the *G. cambogia* extract cytotoxicity, with the effect being comparable to that induced by the extract when tested alone (Figure 2A,D). Similarly, at a concentration of 0.6 µg/mL, montelukast increased the cytotoxicity of *G. cambogia* 100 µg/mL by about 4% (Figure 2C). Conversely, 0.3 µg/mL, 3 µg/mL, and 6 µg/mL of montelukast were highlighted as the most cytotoxic concentrations (Figure 2B,E,F).

Indeed, montelukast was able to increase the inhibition of cell viability induced by *G. cambogia* in a statistically significant way, especially at the highest tested concentrations. In particular, the 0.3 µg/mL concentration raised the cytotoxicity of 250 µg/mL and 500 µg/mL of *G. cambogia* extract by about 13% and 21%, respectively. However, a slight increase (from 4% to 8%) was already noticeable at the lowest tested concentrations (Figure 2B). At 3 µg/mL, montelukast already increased the *G. cambogia* cytotoxicity at the concentration of 1 µg/mL, reaching maximum effects of 20% and 30% at 250 µg/mL and 500 µg/mL, respectively (Figure 2E). Finally, montelukast 6 µg/mL determined a rise in *G. cambogia* cytotoxicity of about 15% and 17% at the concentrations of 1 µg/mL and 10 µg/mL. The maximum effect was reached at the concentration of 250 µg/mL with a reduction in cell viability of about 21%. Conversely, only a slight, although statistically significant, increase in the cytotoxicity was observed at the highest tested concentration of 500 µg/mL (8% reduction in cell viability) (Figure 2F).

**Figure 3:**
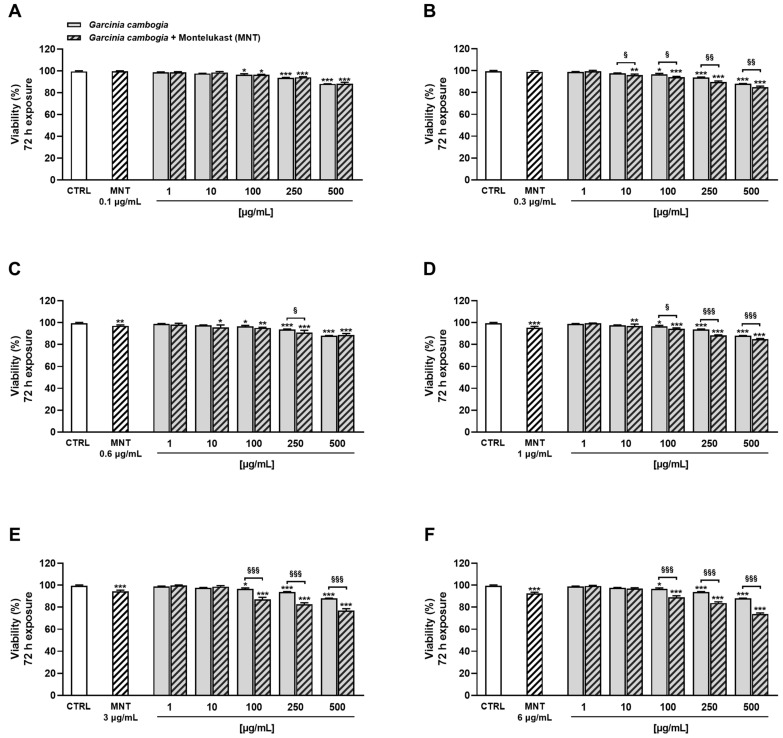
Cytotoxicity of Garcinia cambogia standardized extract in combination with montelukast 0.1 μg/mL (**A**), 0.3 μg/mL (**B**), 0.6 μg/mL (**C**), 1 μg/mL (**D**), 3 μg/mL (**E**), and 6 μg/mL (**F**) in human intrahepatic cholangiocytes H69 determined by MTT assay after 72 h of exposure. Values are expressed as mean ± SEM from at least three independent experiments (n = 3). * *p* < 0.05, ** *p* < 0.01, and *** *p* < 0.001 (ANOVA + Dunnett’s multiple comparison post-test), significantly lower with respect to CTRL. ^§^ *p* < 0.05, ^§§^ *p* < 0.01, and ^§§§^ *p* < 0.001 (Student’s *t*-test), significantly lower with respect to the corresponding concentration of Garcinia cambogia standardized extract alone.

In addition, under the 72 h exposure protocol, montelukast was able to enhance *G. cambogia* cytotoxicity, except for the 0.1 µg/mL concentration (Figure 3A).

Combining the *G. cambogia* extract with 0.3 µg/mL, 0.6 µg/mL, and 1 µg/mL montelukast, inhibition rises in cell viability ranging from 2% to 5% were highlighted (Figure 3B–D). Conversely, a higher effect was observed in combination with both 3 µg/mL and 6 µg/mL montelukast concentrations, reaching a maximum increase in *G. cambogia* cytotoxicity of about 14% (Figure 3E,F).

**Figure 4:**
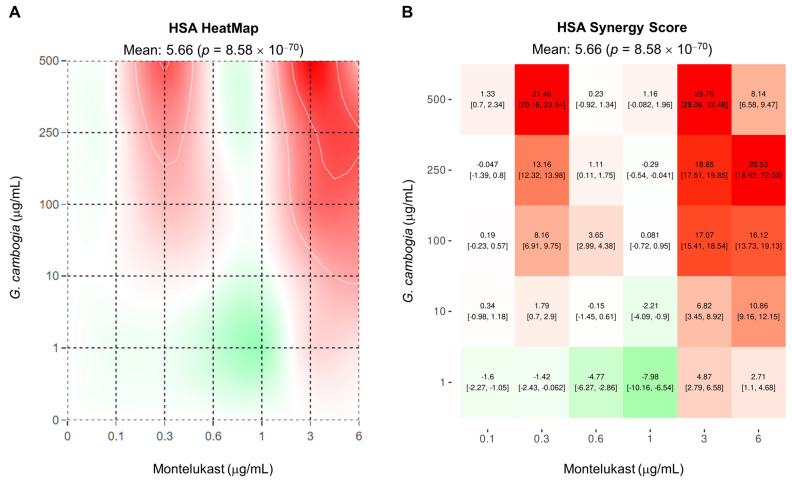
Type of interaction between montelukast and Garcinia cambogia after treatment of normal cholangiocytes H69 for 24 h. (**A**) Heat map and (**B**) synergy score distribution obtained by the HSA reference model. Synergy score interpretation: <−10, the interaction between two drugs is likely to be antagonistic (green shades); −10 to 10, the interaction between two drugs is likely to be additive (from light green to light red shades); >10, the interaction between two drugs is likely to be synergistic (red shades) [40].

On the basis of the obtained results, montelukast displayed potentiation effects towards *G. cambogia*-induced cytotoxicity, especially under the 24 h treatment. Therefore, the abovementioned time exposure was chosen to characterize the nature of the interaction between the two tested samples. To this aim, the web-free application SynergyFinder Plus, which allows drug combination data to be analyzed, was exploited [40]. In particular, the Highest Single Agent (HSA) reference model [45] was used considering that montelukast alone was ineffective at almost all concentrations. Overall, the analysis of the cell viability data after 24 h exposure to the combination of montelukast with the *G. cambogia* extract highlighted an additive effect, as shown in Figure 4.

Both the heat map chart and the synergy score matrix highlighted a prevalence of white-reddish shades with respect to the greenish ones (Figure 4A,B).

The total synergy score of the combination using the HCA method was 5.66; thus, it was concluded that the combination was additive. However, synergistic areas were also evidenced in both graphs. In particular, synergism was highlighted by combining montelukast 0.3 µg/mL, 3 µg/mL, and 6 µg/mL with *G. cambogia* in the concentration range of 10–500 μg/mL.

#### 3.2.3. Montelukast Increases *G. cambogia* Cytotoxicity by Enhancing Its Oxidative Properties

Considering the role played by oxidative stress in the induction of liver and bile duct injuries [10], the levels of reactive oxygen species (ROS) were determined by DCFH-DA assay.

**Figure 5:**
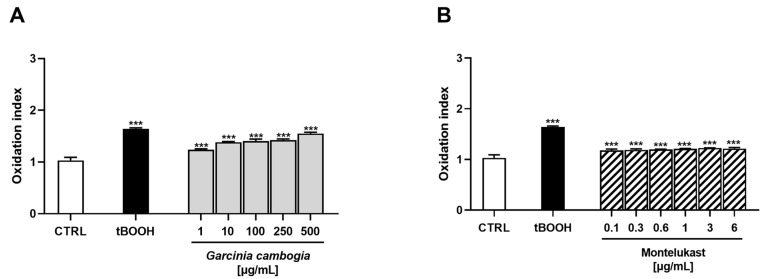
Effect of Garcinia cambogia standardized extract (**A**) and montelukast (**B**) on the ROS levels in human intrahepatic cholangiocytes H69 determined by 2,7-dichlorofluorescein diacetate assay. A protocol consisting of a 24 h treatment with the samples was applied. Conversely, a 3 h exposure schedule was used for the positive control tert-butyl hydroperoxide (tBOOH; 500 μM) due to its marked oxidative properties [38]. ROS levels are expressed as oxidation index with respect to the basal levels. Data are mean ± SE from at least three independent experiments (n = 3). *** *p* < 0.001 (one-way ANOVA followed by Dunnett’s multiple comparison post-test) vs. CTRL.

Under our experimental conditions, the *G. cambogia* extract induced a significant and dose-dependent increase in oxidative stress (Figure 5A). Indeed, already at the lowest tested concentration (1 µg/mL), a 1.2-fold rise in intracellular ROS levels with respect to the control was observed. The maximum effect was reached at 500 µg/mL, with a 1.5-fold increase in ROS production (Figure 5A). Conversely, montelukast exhibited only slight, albeit statistically significant, oxidative properties. However, no differences were found among the tested concentrations (Figure 5B). As expected, the oxidative agent tBOOH induced marked ROS production (about a 1.6-fold increase with respect to the control) (Figure 5).

**Figure 6:**
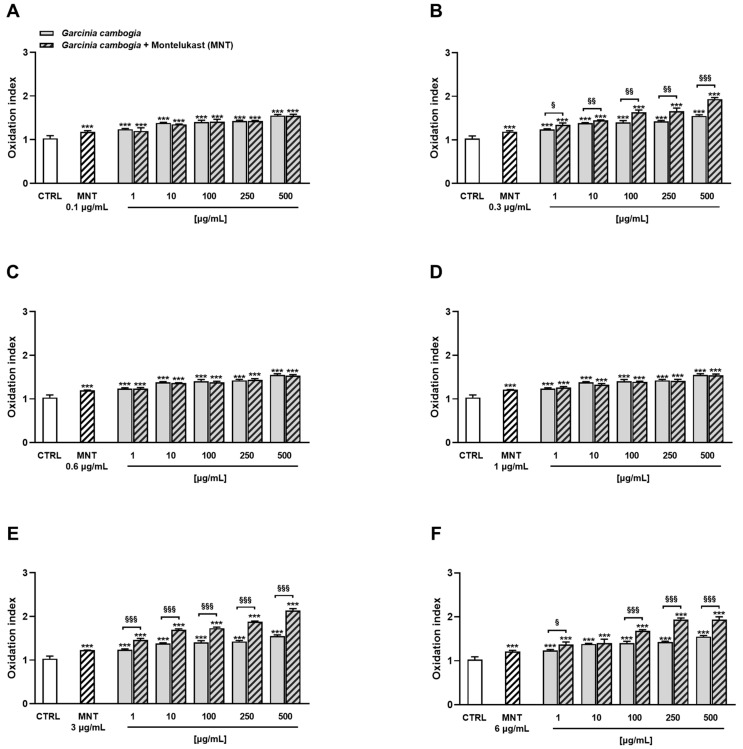
Effect of the combination of Garcinia cambogia standardized extract with montelukast 0.1 μg/mL (**A**), 0.3 μg/mL (**B**), 0.6 μg/mL (**C**), 1 μg/mL (**D**), 3 μg/mL (**E**), and 6 μg/mL (**F**) on the ROS levels in human intrahepatic cholangiocytes H69 determined by 2,7-dichlorofluorescein diacetate assay. ROS levels are expressed as oxidation index with respect to the basal levels. Data are mean ± SE from at least three independent experiments (n = 3). *** *p* < 0.001 (one-way ANOVA followed by Dunnett’s multiple comparison post-test) vs. CTRL. ^§^ *p* < 0.05, ^§§^ *p* < 0.01, and ^§§§^ *p* < 0.001 (Student’s *t*-test), significantly lower with respect to the corresponding concentration of Garcinia cambogia standardized extract alone.

Although harmless when tested alone, montelukast was able to increase the oxidative stress induced by *G. cambogia* extract (Figure 6). Analogously to the cytotoxicity experiments, the concentrations of 0.1 µg/mL, 0.6 µg/mL, and 1 µg/mL were ineffective (Figure 6A,C,D).

**Figure 7:**
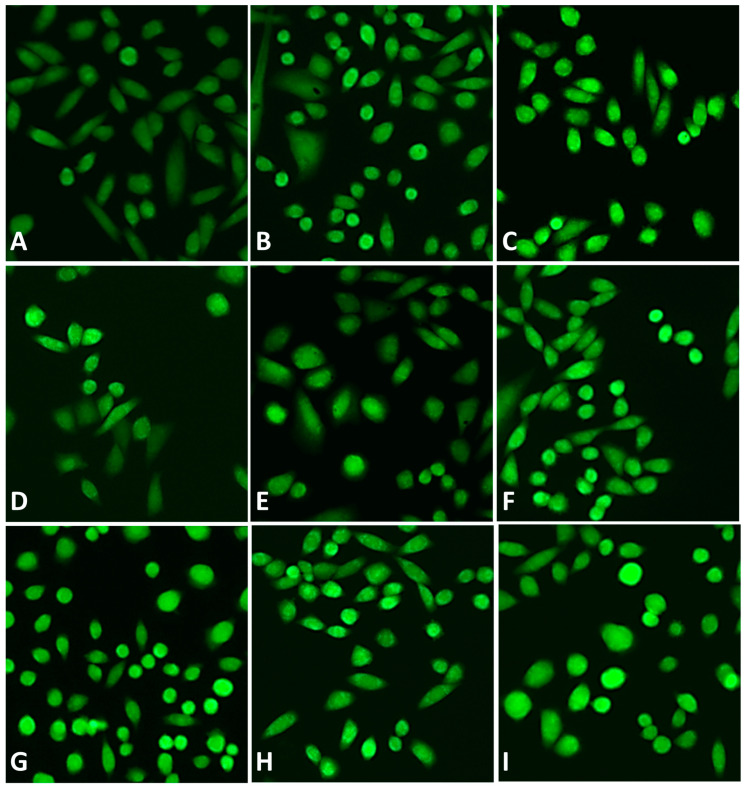
Representative images of intracellular ROS after treatment of H69 normal cholangiocytes with tested samples. (**A**) Vehicle; (**B**) montelukast 3 μg/mL; (**C**) tBOOH 500 μM; (**D**) G. cambogia 100 µg/mL; (**E**) G. cambogia 250 µg/mL; (**F**) G. cambogia 500 µg/mL; (**G**) G. cambogia 100 µg/mL + montelukast 3 μg/mL; (**H**) G. cambogia 250 µg/mL + montelukast 3 μg/mL; (**I**) G. cambogia 500 µg/mL + montelukast 3 μg/mL. Original magnification 10×.

The combination of *G. cambogia* with montelukast 0.3 μg/mL, 3 μg/mL, and 6 μg/mL resulted in a statistically significant rise in ROS production (Figure 6B,E,F). In particular, a maximum 1.2-fold increase was achieved by combining *G. cambogia* with montelukast 0.3 μg/mL. Instead, the 3 μg/mL and 6 μg/mL concentrations of the drug further raised ROS production (by about 1.4-fold with respect to the extract alone) in combination with 250 μg/mL and 500 μg/mL *G. cambogia* extract, respectively. Figure 7 shows some representative images of intracellular ROS after treatment of H69 cells with the 100 μg/mL, 250 μg/mL, and 500 μg/mL *G. cambogia* extract and 3 μg/mL montelukast, both alone or in combination.

#### 3.2.4. *G. cambogia* and Montelukast Affect Nrf2 Cytoplasmic Expression

Considering the ability of *G. cambogia* extract to induce oxidative stress, especially in the presence of montelukast 3 µg/mL, in the subsequent experiments, we explored the possible modulation of the cytoplasmic expression of Nrf2, a known transcription factor that plays an important role in the cellular defense by regulating the adaptive response to oxidative stress [29].

**Figure 8:**
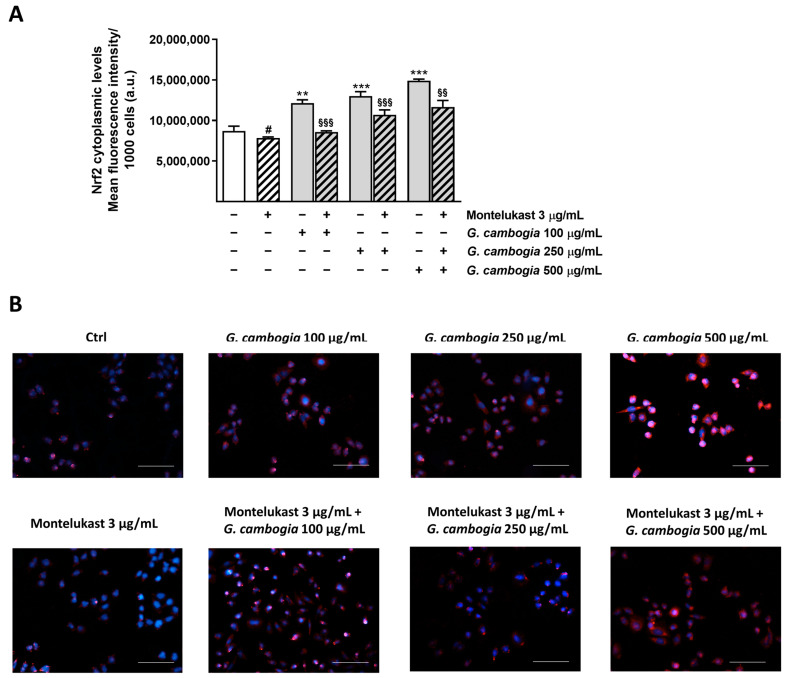
Effects of G. cambogia extract (100, 250 and 500 µg/mL), montelukast (3 µg/mL) and their combinations on the Nrf2 cytoplasmic expression in H69 cells. (**A**) Data displayed as mean ± SE of at least two independent experiments with at least three technical replicates (n = 6). (**B**) Representative images of cells stained by suitable antibodies and Hoechst 33258 dye. # *p* < 0.05, significant difference of montelukast with respect to Ctrl (Student’s *t*-test). ** *p* < 0.01 and *** *p* < 0.001, significant difference of G. cambogia extract with respect to Ctrl (ANOVA followed by Dunnett’s multiple comparison post-test). ^§§^ *p* < 0.01 and ^§§§^ *p* < 0.001, significant difference of G. cambogia extract + montelukast with respect to G. cambogia extract at the corresponding concentration (ANOVA followed by Dunnett’s multiple comparison post-test). Scale bars = 40 µm.

Under our experimental conditions, montelukast (3 µg/mL) significantly affected the basal cytoplasmic levels of Nrf2, by reducing its expression by about 18% (Figure 8A,B). Conversely, 100 µg/mL, 250 µg/mL, and 500 µg/mL *G. cambogia* extract induced about a 27%, 36%, and 56% increase in the Nrf2 expression with respect to the control, respectively. When combined with montelukast, a Nrf2 decrease with respect to *G. cambogia* extract alone was observed. Indeed, its cytoplasmic expression was reduced by about 38%, 20%, and 28% by the combination of montelukast and 100 µg/mL, 250 µg/mL, and 500 µg/mL *G. cambogia* extract concentrations, respectively (Figure 8A,B).

## 4. Discussion

Recently, the prevalence of overweight and obesity has increased worldwide, reaching pandemic proportions [46]. The main causes of their occurrence are represented by a reduction in physical activity, an increase in sedentary lifestyles, a higher consumption of low-cost fast foods, and low adherence to healthy diets [47]. Obesity is associated with several diseases, including cardiovascular, diabetes, hypertension, hyperlipidemia, and hepatic ones, thus representing an important issue for public health and a great burden for healthcare systems [46]. Therefore, there has been a continuous search for new strategies to reduce body weight. Several drugs have been introduced in the market; however, some of them have been withdrawn (e.g., fenfluramine, dexfenfluramine, sibutramine) due to their side effects [48]. At present, only orlistat, naltrexone/bupropion, and liraglutide have been approved for long-term use by the European Medicine Agency (EMA) and the US Food and Drug Administration (FDA), although these drugs are also not devoid of side effects [49]. Therefore, people often resort to alternative strategies to reduce body weight, such as the use of botanical supplements, under the misconception that natural is a synonym of safe. However, several reports of adverse events associated with the consumption of herbal supplements have been highlighted [15,16,48].

Among them, recently, a growing number of hepatotoxicity cases associated with the use of *G. cambogia*-containing supplements have been reported [18,19,26,27], thus supporting our interest in performing an in-depth study of the cases that have occurred in Italy and in investigating the potential mechanisms triggering liver damage.

In the present study, we evaluated the toxicity risk of *G. cambogia* by first taking into account the suspected hepatotoxic reactions collected within the database of the IPS and then exploring in vitro the oxidative stress and cell defence impairments as possible underpinning mechanisms of liver injury.

From March 2002 to March 2022, the IPS collected eight reports of hepatic adverse reactions related to the use of *G. cambogia* for weight loss, which exclusively involved women and were mostly severe. The reports 1 (ID 275), 3 (ID 1080), 4 (ID 1382), 5 (ID 1389), and 6 (ID 1415) were previously published by Actis et al. [35] and Crescioli et al. [22] but are included in the present manuscript to give a complete picture of the Italian cases. Overall, a small number of cases was highlighted; however, it is important to outline that one of the major limitation of spontaneous reports is the underreporting due to the underestimation of the potential risk associated with natural products’ consumption [36]. In spite of this, several other cases have been described in the literature. In particular, Crescioli et al. [22] retrieved 32 articles, for a total of 66 patients, describing adverse reactions related to *G. cambogia* consumption.

Acute liver injury, liver failure, and hepatotoxicity were reported in 17 out of 32 studies and involved 50 patients who consumed *G. cambogia* dietary supplements or *G. cambogia* pure extract. The treatment duration and latency of the reaction were variable ranging from a few days to more than one year. According to our findings, women were mostly involved (62%), likely due to their greater use of food supplements [50].

Overall, the cases outlined within the present study and those reported in the literature share some common pattern of symptoms, including abdominal pain, vomiting, nausea, fatigue, and alterations in liver parameters such as transaminases, alkaline phosphatase, and bilirubin. Notably, in most cases, the withdrawal of the *G. cambogia* supplement determined an improvement in the symptoms and a normalization of the altered parameters. Nevertheless, we are aware that *G. cambogia* supplements often contain a considerable number of components, among which are some whose hepatotoxicity is well known (e.g., green tea, turmeric) [15,16]. Moreover, the presence of several ingredients can increase the probability of interactions with concomitant pharmacological treatment [36]. However, liver injury cases have also been associated with supplements in which *G. cambogia* represented the only ingredient. Therefore, it is of outmost importance to first understand its safety profile when used alone or combined with a single drug in order to subsequently investigate its role in multicomponent products or in combination with several drugs.

For these reasons, we implemented a toxicological in vitro model aimed at verifying, and possibly confirming, the onset of liver toxicity at the cellular level induced by *G. cambogia*. To perform the experiments, a dry extract of the fruit standardized to contain 60% of hydroxycitric acid was exploited, considering that it is the most common among the commercially available supplements. Moreover, in order to explore a possible herb–drug interaction, combination experiments with the anti-asthmatic drug montelukast were performed, considering the first report (ID 275) collected within the IPS. Indeed, many people use botanicals simultaneously with conventional drugs, under the misconception that remedies of natural origin are safe. However, plants contain several phytochemicals, each of which can interfere with prescribed medications, thus causing potentially harmful side effects or leading to the loss of or decreased therapeutic benefits of the drugs. This issue is of particular relevance for patients affected by chronic disease and for the elderly population who are also taking multiple prescription medications to manage their concurrent conditions [51]. Therefore, considering the widespread use of botanicals in modern society, it is crucial to identify potential herb–drug interactions in order to minimize the associated risks and ensure public health.

Under our experimental conditions, *G. cambogia* showed a low cytotoxicity in the range of concentration tested, reaching a maximum effect of about 25%. To the best of our knowledge, this is the first study to have investigated the toxicity of *G. cambogia* in hepatic cells. However, the lack of toxicity has also been reported in cultured nasal epithelial cells and 3T3-L1 adipocytes at a concentration range within 0.012–5 mg/mL [52,53]. Moreover, some evidence exists for the pure compound garcinol, a polyisoprenylated benzophenone isolated from *Garcinia* spp., which has been reported to exert hepatoprotective properties by supporting hepatocyte survival through the suppression of apoptosis [54]. Conversely, several in vivo experiments have been carried out showing a good safety profile for both *G. cambogia* and HCA and a protective effect on the liver [17].

As expected, an increase in the cytotoxicity was observed in the presence of montelukast reaching a maximum effect of 55%. In particular, the type of the interaction between the two samples was considered additive, although at specific dose combinations, synergism was detected. This effect could be due to the ratio between the combined agents [55]. Indeed, it was previously reported that a cell line may be less susceptible to a specific drug combination presented at a certain ratio, while being highly affected by exposure to the same drugs but at a different ratio. It is likely that the deep interconnection between biochemical pathways and cellular biology is responsible for the observed effect [56].

Regarding montelukast, it is an orally active compound used in asthmatic inflammation. It is generally considered a safe drug, with the overall incidence of adverse events being comparable to a placebo [57]. However, it has been linked to cases of liver injuries [34,35]. Under our experimental conditions, montelukast did not affect H69 cell viability when tested alone. However, it has been reported to induce severe cytotoxicity, by acting at a mitochondrial level, in the human HepaRG hepatic cell line, although at concentrations 10 times higher than those tested within our work [58].

Moreover, contrary to what we observed in combination with *G. cambogia*, other studies ascribed protective properties against the liver damage induced by other drugs to montelukast. Indeed, Fei et al. [59] demonstrated that montelukast exerted a protective action against pemetrexed-induced cytotoxicity in primary human LO-2 hepatocytes by mitigating endoplasmic reticulum (ER) stress and nucleotide oligomerization domain-like receptor protein 3 (NLRP3) inflammasome activation. Moreover, it has been highlighted by in vitro and in vivo experiments that montelukast is able to counteract acetaminophen-induced hepatotoxicity through the upregulation of the hepatic GSH/GSSG system and the suppression of oxidative stress [60]. Although contrasting results were obtained within our experimental model, it should be outlined that unlike the abovementioned studies in which montelukast was tested in combination with another single drug, *G. cambogia* extract contains hundreds of compounds that could interfere with several intracellular pathways, thus making the picture more complex.

In light of this evidence, we decided to investigate the modulation of redox homeostasis induced by both *G. cambogia* and montelukast. While a slight increase in ROS release was observed for the anti-asthmatic drug, *G. cambogia* determined an increase in oxidative stress comparable to that of the positive control. Intriguingly, their combination greatly affected the ROS homeostasis by inducing an oxidative effect higher than that of tBOOH.

In accord with our results, Kim and coworkers highlighted that the treatment with *G. cambogia* (60% HCA) of rats fed with a high-fat diet triggered oxidative stress, inflammation, and liver fibrosis [32]. However, other studies support an antioxidant role for both *G. cambogia* and montelukast. In particular, regarding the latter, it has been shown that it determined a decrease in ROS levels in the livers of C57BL/6J mice treated with acetaminophen [60]. Moreover, it was also able to prevent the induction of oxidative stress by decreasing hepatic MDA content and enhancing GSH levels [61,62].

Similar results were also observed for *G. cambogia.* Indeed, Sripradha et al. [63] found that an ethanolic extract of this plant significantly increased the liver antioxidant defenses in rats fed with a fat-rich diet. Accordingly, another study showed a normalization of GSH levels in adult male Wistar rats fed with a high-fat fructose diet after treatment with *G. cambogia* extract (60% HCA) [64]. Moreover, Han et al. [65] observed that *G. cambogia* (60% HCA) was able to activate the intracellular antioxidant defense system, thus reducing ROS production in HepG2 cells. These effects were ascribed to the content of HCA, according to other studies [65,66,67]. Despite this evidence, no studies have investigated, until now, the effect of *G. cambogia* and conventional drug combination in the modulation of redox homeostasis. Therefore, our findings highlight the importance of improved investigation of this mechanism as a potential trigger of hepatic adverse reaction.

To this aim, we investigated the modulation of Nrf2 expression, obtaining results in agreement with published evidence. Indeed, Jung et al. [68] highlighted that montelukast raised the nuclear expression of Nrf2 while lowering the cytoplasmic one, in a model of allergic airway inflammation. Moreover, an increase in the Nrf2 mRNA expression and nuclear translocation were observed after treatment with *G. cambogia* extract (60% HCA) [64,65]. However, also in this case, there are no studies in the literature aimed at investigating the effect of *G. cambogia* in combination with conventional drugs on the Nrf2 pathway. Notably, under our experimental conditions, the increase in Nrf2 expression corresponded to a rise in cell cytotoxicity. Usually, Nrf2 is activated as an adaptive defense response to enhance cell survival and protect the cell against oxidative-stress-induced apoptosis [69]. In addition to this, it has been shown that, in cholangiocytes, the activation of Nrf2 induces a reduction in the type 3 isoform of the inositol 1,4,5-trisphosphate receptor (ITPR3), the most abundant intracellular calcium release channel in cholangiocytes, required for bicarbonate secretion by bile ducts. Interestingly, the reduced expression of ITPR3 is often observed in patients with cholestatic disorders [69].

In conclusion, our results highlight that *G. cambogia* is able to induce hepatic oxidative stress, albeit with a mild cytotoxicity. We hypothesize that under normal conditions, as also confirmed in our experimental model, the cellular antioxidant defense systems are able to counteract the *G. cambogia* oxidative damage, thus allowing cell survival. This could explain the small number of *G. cambogia* hepatoxicity cases found in the IPS and in the literature. However, although harmless in healthy subjects, *G. cambogia* could determine the occurrence of hepatic adverse reactions in more susceptible people due, for instance, to the presence of HLA-B*35:01 polymorphism [70], or concomitant pathologies, such as obesity, or under pharmacological treatment. In particular, growing evidence has shown that obese patients present an altered antioxidant status and an excessive production of ROS [71]; moreover, pharmacological therapy could increase oxidative stress [72]. Under these conditions, the concomitant consumption of *G. cambogia* supplements could further worsen the oxidative imbalance, thus triggering the occurrence of adverse reactions. In conclusion, our findings substantiate safety concerns regarding *G. cambogia*-containing supplements and provide insights into potential mechanisms underlying liver injury associated with *G. cambogia* consumption and drug interactions. Nevertheless, further research is required to fully elucidate these aspects.

## Figures and Tables

**Table 1:** Liver adverse reactions related to *Garcinia-cambogia*-containing supplements.

Gender/ Age n/ID References	Adverse Reaction	Product	Reason for Use	Latency/Treatment Duration (Days)	Laboratory Data ^a^	Seriousness	Other Medications/ Other Diseases	Outcome	Dechallenge/Rechallenge
F/45 1/275 [35]	Massive hepatic necrosis, acute hepatic failure	1. Top line advantra Z 2. Kalo rapido	Weight loss	1. 15/8 2. NR/NR	AST > 1000 ALT 2512 TB 5.7 GGT 31	H	Montelukast/Hepatic steatosis	D	1. NR/NR 2. NR/NR
F/39 2/309	Acute hepatitis	1. Peso stop 2. Magrixs	Weight loss	1. 55/54 2. 213/522	AST-ALT > 3000, Bilirubin 30 Virus markers: negative	H	NR/NR	CR	1. +/NR 2. +/NR
F/39 3/1080 [22]	Cholestatic hepatitis	1. Obless 2. *Citrus aurantium* 6%, Rhodiola, Orthosiphon	Weight loss	1. 40/30 2. 40/30	AST 1682 ALT 1868 TB 14.79 DB 13.17	H	Metildopa/Metabolic syndrome	P	1. NR/NR 2. NR/NR
F/61 4/1382 [22]	Cholestatic hepatitis	Super ananas slim	Weight loss	NR/NR	AST 1071 ALT 1625	H	NR/NR	CR	+/NR
F/52 5/1389 [22]	Acute hepatitis	1. *G. cambogia* 2. Green coffee	Weight loss	1. NR/NR 2. NR/NR	AST 1442 ALT 1819 TB 14.7 GGT 223 Virus markers: negative	NR	NO/NR	CR	1. +/NR 2. +/NR
F/47 6/1415 [22]	Abdominal pain, hepatomegaly, acute hepatitis	Herbalife, thermo giallo	Weight loss	NR/NR	AST 67 ALT 266 TB 0.7	H	Levothyroxine/Hypertension, hypothyroidism, obesity	CR	+/NR
F/57 7/1853	Acute hepatitis	1. CRCMN-P 2. Garcinia 1000 3. Piperina and Curcuma	Weight loss	1. 30/NR 2. 30/NR 3. 15/15	ALT 2291 TB 7.5	H	Eliquis, Almarytm, Eutirox, Lasix /Hypothyroidism, atrial fibrillation	R	1. +/NR 2. +/NR 3. +/NR
F/42 8/1988	Jaundice, asthenia, nausea anorexia	Garcinia body slim	NR	NR/NR	ALT 2388 TB 3.5 GGT 341	H	NR/Papillary thyroid carcinoma with hypothyroidism; bronchial asthma	R	NR/NR

^a^ Normal values: AST 8–48 U/L; ALT 7–55 U/L; total bilirubin 0.2–1.1 mg/dL; direct bilirubin < 0.3 mg/dL; GGT 38–55 U/L. F: female; NR: not reported; H: hospitalization; TB: total bilirubin; DB: direct bilirubin; +: positive; D: death; CR: complete resolution; R: recovering; P: persistent.

**Table 2:** Composition of suspected products without excipients.

n/ID	Product Name	Composition
1/275	1. Top line advantra Z	1. Advantra Z^TM^ *Citrus aurantium* fruit peel d.e. (dry extract) 6% synephrine; *Garcinia cambogia* fruit peel d.e. 60% hydroxycitric acid; L-phenylalanine; *Gymnema silvestris* leaves d.e. 25% gymnemic acid; L-carnitine hydrochloride; *Phaseolus vulgaris* bean pod d.e.
	2. Kalo rapido	2. Hydrolyzed collagen; sodium hyaluronate; *Betula alba* leaf extract; *Citrus aurantium* leaf extract; *Magnolia officinalis* bark extract; *Bambusa vulgaris* leaf extract; glutamine; L-orthine; magnesium pyruvate.
2/309	1. Peso stop	1. Green tea leaves d.e. 5% caffeine; *Gymnema silvestre* leaves d.e. 26% gymnemic acids; bean protein concentrate (phaseolamine); pineapple fruit d.e. enzymatic activity 250 GDU/g; Clarinol natural concentrate of omega 6 fatty acids; conjugated linoleic acid (CLA) from vegetable oils; choline bitartrate; inositol; L-carnitine; cinnamon bark d.e. 1.6% MHCP (methylhydroxycalcalone polymer); green coffee seed d.e. 50% chlorogenic acid; bioperine (*Piper nigrum*) fruit d.e. 95% in piperine.
	2. Magrixs	2. Thermal water from the Montegrimano spa; green tea (*Camellia sinensis*) leaves; Garcinia (*Garcinia cambogia*) fruit; pineapple (*Ananas sativa*) stem; fennel (*Foeniculum vulgare*) seeds; orthosiphon (*Ortosiphon stamineus*) leaves; burdock (*Arctium lappa*) root; *Cassia nomame* (*Cassia mimosoides*) plant d.e. 8% (2s)-3,4,7-trihydroxyflavan-(4 beta − 8) catechin; banaba (*Lagerstroemia speciosa*) leaves d.e. 1% corosolic acid.
3/1080	1. Obless	1. Citrus (*Citrus aurantium* var. *amara* L., fruit) d.e. 10% synephrine (equal to a content of 14 mg) 140 mg; Garcinia (*Garcinia cambogia* Desr., fruit) d.e. 60% hydroxycitric acid (equal to a content of 72 mg) 120 mg; Ortosifon (*Orthosiphon stamineus* Benth, leaves) d.e. 0.2% sinensetin (equal to a content of 0.2 mg.) 100 mg; *Griffonia simplicifolia* Baill., seeds d.e. 99% 5-hydroxy-L-tryptophan (equal to a content of 75 mg) 75 mg.
	2. *Citrus aurantium* 6%, rhodiola, orthosiphon	2. *Citrus aurantium* 6%; rhodiola; orthosiphon.
4/1382	Super ananas slim	Pineapple concentrated juice (*Ananas comosus* (L.) Merr.) fruit; pineapple (*Ananas comosus* (L.) Merr.) stem d.e. 250 GDU/g bromelain; mate (*Ilex paraguariensis* A. St. Hill.) leaf d.e. 2% caffeine; garcinia cambogia (*Garcinia cambogia* (gaernt) Desr.) fruit d.e. 60% hydroxycitric acid; pineapple (*Ananas comosus* (L.) Merr.) stem d.e. 2500 GDU/g bromelain.
5/1389	1. *Garcinia cambogia* 2. Green coffee	1. *Garcinia cambogia* extract 400 mg 60% hydroxycitric acid (240 mg). 2. Green Coffee extract 400 mg 50% chlorogenic acid (200 mg).
6/1415	Herbalife, thermo giallo	*Garcinia cambogia* extract; chromium chloride.
7/1853	1. CRCMN-P 2. Garcinia 1000 3. Piperina and Curcuma	1. Unknown composition 2. Garcinia not otherwise specified 3. Turmeric (*Curcuma longa* root, 360 mg) d.e. 95% curcuminoids; turmeric (*Curcuma longa* root powder, 360 mg); black pepper (*Piper nigrum* fruit, 80 mg) d.e. 95% piperine.
8/1988	Garcinia body slim	Inulin 500 mg; garcinia fruit d.e. 300 mg of which 180 mg hydroxycitric acid; green tea 20 mg; coleus d.e. 20 mg; gymnema d.e.

## Data Availability

Data is contained within the article and supplementary material.

## Supplementary Materials

The following supporting information can be downloaded at: https://www.mdpi.com/article/10.3390/antiox12091771/s1, Figure S1: Technical data sheet of the *Garcinia cambogia* fruit standardized extract.
