# Supplementary material for: Interaction of Garcinia cambogia (Gaertn.) Desr. and Drugs as a Possible Mechanism of Liver Injury: The Case of Montelukast
Source: Antioxidants (Basel). 2023 Sep 16;12(9):1771. doi: 10.3390/antiox12091771 (PMC10525400; doi:10.3390/antiox12091771)
Supplement: Supplementary file 1 [file antioxidants-12-01771-s001.zip › antioxidants-2579396-supplementary.pdf]

antioxidants

Supplementary material

Interaction of *Garcinia cambogia* (Gaertn.) Desr. and Drugs as a Possible Mechanism of Liver Injury: The Case of Montelukast

Silvia Di Giacomo [1,2,*], Antonella Di Sotto [2,*], Ester Percaccio [2], Erica Scuotto [2], Cecilia Battistelli [3], Gabriela Mazzanti [2], Francesca Menniti-Ippolito [4] and Ilaria Ippoliti [4]

1 Department of Food Safety, Nutrition and Veterinary Public Health, National Institute of Health, Viale Regina Elena 299, 00161 Rome, Italy
2 Department of Physiology and Pharmacology "V. Erspamer", Sapienza University of Rome, P.le Aldo Moro 5, 00185 Rome, Italy
3 Department of Molecular Medicine, Sapienza University of Rome, Viale Regina Elena 324, 00161 Rome, Italy
4 National Centre for Drug Research and Evaluation, National Institute of Health, Viale Regina Elena 299, 00161 Rome, Italy
* Correspondence: silvia.digiacomo@iss.it (S.D.G.); antonella.disotto@uniroma1.it (A.D.S.)

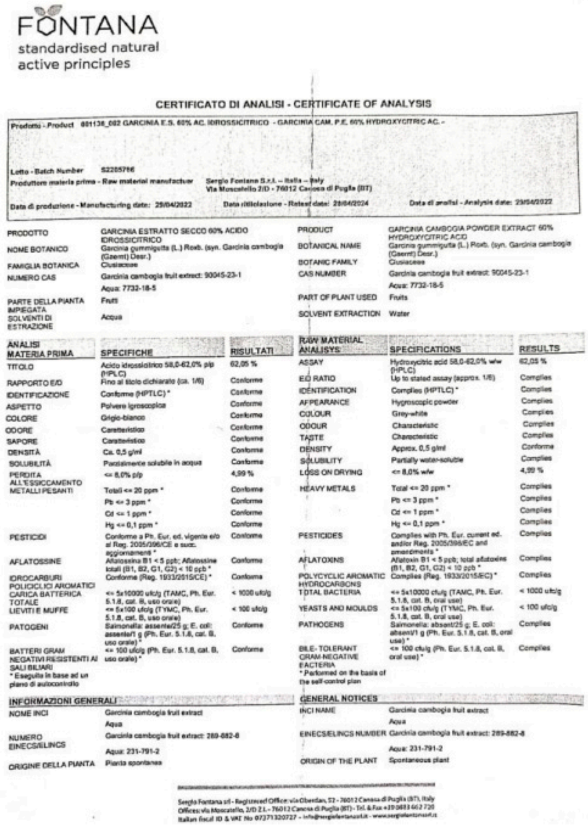

FONTANA
standardised natural active principles

CERTIFICATO DI ANALISI - CERTIFICATE OF ANALYSIS

Prodotto - Product 001138_002 GARCINIA E.S. 60% AC. IDROSSICITRICO - GARCINIA CAM. P.E. 60% HYDROXYCITRIC AC. -

Lotto - Batch Number S2205766
Produttore materia prima - Raw material manufactuer: Sergio Fontana S.r.l. – Italia – Italy, Via Moscatello 2/D - 76012 Canosa di Puglia (BT)
Data di produzione - Manufacturing date: 29/04/2022 Data rititolazione - Retest date: 28/04/2024 Data di analisi - Analysis date: 29/04/2022

PRODOTTO	GARCINIA ESTRATTO SECCO 60% ACIDO IDROSSICITRICO	PRODUCT	GARCINIA CAMBOGIA POWDER EXTRACT 60% HYDROXYCITRIC ACID
NOME BOTANICO	Garcinia gummigutta (L.) Roxb. (syn. Garcinia cambogia (Gaertn) Desr.)	BOTANICAL NAME	Garcinia gummigutta (L.) Roxb. (syn. Garcinia cambogia (Gaertn) Desr.)
FAMIGLIA BOTANICA	Clusiaceae	BOTANIC FAMILY	Clusiaceae
NUMERO CAS	Garcinia cambogia fruit extract: 90045-23-1; Aqua: 7732-18-5	CAS NUMBER	Garcinia cambogia fruit extract: 90045-23-1; Aqua: 7732-18-5
PARTE DELLA PIANTA IMPIEGATA	Frutti	PART OF PLANT USED	Fruits
SOLVENTI DI ESTRAZIONE	Acqua	SOLVENT EXTRACTION	Water

ANALISI MATERIA PRIMA	SPECIFICHE	RISULTATI	RAW MATERIAL ANALISYS	SPECIFICATIONS	RESULTS
TITOLO	Acido idrossicitrico 58,0-62,0% p/p (HPLC)	62,05 %	ASSAY	Hydroxycitric acid 58,0-62,0% w/w (HPLC)	62,05 %
RAPPORTO E/D	Fino al titolo dichiarato (ca. 1/6)	Conforme	E/D RATIO	Up to stated assay (approx. 1/6)	Complies
IDENTIFICAZIONE	Conforme (HPTLC) *	Conforme	IDENTIFICATION	Complies (HPTLC) *	Complies
ASPETTO	Polvere igroscopica	Conforme	APPEARANCE	Hygroscopic powder	Complies
COLORE	Grigio-bianco	Conforme	COLOUR	Grey-white	Complies
ODORE	Caratteristico	Conforme	ODOUR	Characteristic	Complies
SAPORE	Caratteristico	Conforme	TASTE	Characteristic	Complies
DENSITÀ	Ca. 0,5 g/ml	Conforme	DENSITY	Approx. 0,5 g/ml	Conforme
SOLUBILITÀ	Parzialmente solubile in acqua	Conforme	SOLUBILITY	Partially water-soluble	Complies
PERDITA ALL'ESSICCAMENTO	<= 8,0% p/p	4,99 %	LOSS ON DRYING	<= 8,0% w/w	4,99 %
METALLI PESANTI	Totali <= 20 ppm *	Conforme	HEAVY METALS	Total <= 20 ppm *	Complies
	Pb <= 3 ppm *	Conforme		Pb <= 3 ppm *	Complies
	Cd <= 1 ppm *	Conforme		Cd <= 1 ppm *	Complies
	Hg <= 0,1 ppm *	Conforme		Hg <= 0,1 ppm *	Complies
PESTICIDI	Conforme a Ph. Eur. ed. vigente e/o al Reg. 2005/396/CE e succ. aggiornamenti *	Conforme	PESTICIDES	Complies with Ph. Eur. current ed. and/or Reg. 2005/396/EC and amendments *	Complies
AFLATOSSINE	Aflatossina B1 < 5 ppb; Aflatossine totali (B1, B2, G1, G2) < 10 ppb *	Conforme	AFLATOXINS	Aflatoxin B1 < 5 ppb; total aflatoxins (B1, B2, G1, G2) < 10 ppb *	Complies
IDROCARBURI POLICICLICI AROMATICI	Conforme (Reg. 1933/2015/CE) *	Conforme	POLYCYCLIC AROMATIC HYDROCARBONS	Complies (Reg. 1933/2015/EC) *	Complies
CARICA BATTERICA TOTALE	<= 5x10000 ufc/g (TAMC, Ph. Eur. 5.1.8, cat. B, uso orale)	< 1000 ufc/g	TOTAL BACTERIA	<= 5x10000 cfu/g (TAMC, Ph. Eur. 5.1.8, cat. B, oral use)	< 1000 ufc/g
LIEVITI E MUFFE	<= 5x100 ufc/g (TYMC, Ph. Eur. 5.1.8, cat. B, uso orale)	< 100 ufc/g	YEASTS AND MOULDS	<= 5x100 cfu/g (TYMC, Ph. Eur. 5.1.8, cat. B, oral use)	< 100 ufc/g
PATOGENI	Salmonella: assente/25 g; E. coli: assente/1 g (Ph. Eur. 5.1.8, cat. B, uso orale) *	Conforme	PATHOGENS	Salmonella: absent/25 g; E. coli: absent/1 g (Ph. Eur. 5.1.8, cat. B, oral use) *	Complies
BATTERI GRAM NEGATIVI RESISTENTI AI SALI BILIARI	<= 100 ufc/g (Ph. Eur. 5.1.8, cat. B, uso orale) *	Conforme	BILE-TOLERANT GRAM-NEGATIVE BACTERIA	<= 100 cfu/g (Ph. Eur. 5.1.8, cat. B, oral use) *	Complies

* Eseguita in base ad un piano di autocontrollo / * Performed on the basis of the self-control plan

INFORMAZIONI GENERALI		GENERAL NOTICES	
NOME INCI	Garcinia cambogia fruit extract; Aqua	INCI NAME	Garcinia cambogia fruit extract; Aqua
NUMERO EINECS/ELINCS	Garcinia cambogia fruit extract: 289-882-8; Aqua: 231-791-2	EINECS/ELINCS NUMBER	Garcinia cambogia fruit extract: 289-882-8; Aqua: 231-791-2
ORIGINE DELLA PIANTA	Pianta spontanea	ORIGIN OF THE PLANT	Spontaneous plant

Sergio Fontana srl - Registered Office: via Oberdan, 52 - 76012 Canosa di Puglia (BT), Italy
Offices: via Moscatello, 2/D Z.I. - 76012 Canosa di Puglia (BT) - Tel. & Fax +39 0883 662 720
Italian fiscal ID & VAT No 07271320727 - info@sergiofontanasrl.it - www.sergiofontanasrl.it

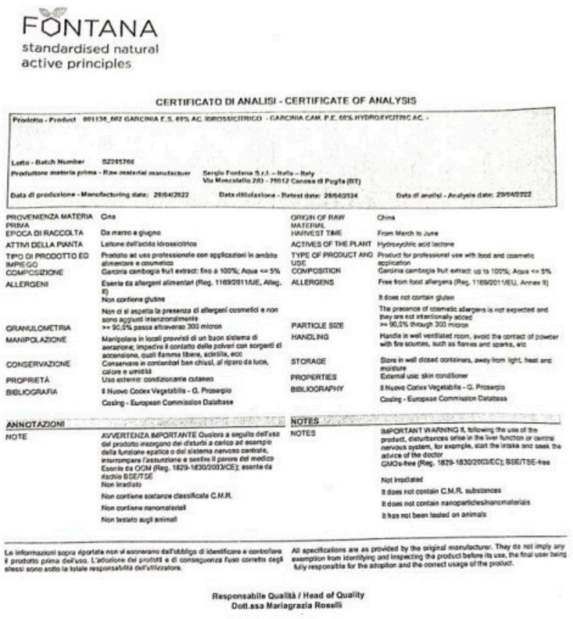

FONTANA
standardised natural active principles

CERTIFICATO DI ANALISI - CERTIFICATE OF ANALYSIS

Prodotto - Product 001138_002 GARCINIA E.S. 60% AC. IDROSSICITRICO - GARCINIA CAM. P.E. 60% HYDROXYCITRIC AC. -

Lotto - Batch Number S2205766
Produttore materia prima - Raw material manufactuer: Sergio Fontana S.r.l. – Italia – Italy, Via Moscatello 2/D - 76012 Canosa di Puglia (BT)
Data di produzione - Manufacturing date: 29/04/2022 Data rititolazione - Retest date: 28/04/2024 Data di analisi - Analysis date: 29/04/2022

PROVENIENZA MATERIA PRIMA	Cina	ORIGIN OF RAW MATERIAL	China
EPOCA DI RACCOLTA	Da marzo a giugno	HARVEST TIME	From March to June
ATTIVI DELLA PIANTA	Lattone dell'acido idrossicitrico	ACTIVES OF THE PLANT	Hydroxycitric acid lactone
TIPO DI PRODOTTO ED IMPIEGO	Prodotto ad uso professionale con applicazioni in ambito alimentare e cosmetico	TYPE OF PRODUCT AND USE	Product for professional use with food and cosmetic application
COMPOSIZIONE	Garcinia cambogia fruit extract: fino a 100%; Aqua <= 5%	COMPOSITION	Garcinia cambogia fruit extract: up to 100%; Aqua <= 5%
ALLERGENI	Esente da allergeni alimentari (Reg. 1169/2011/UE, Alleg. II); Non contiene glutine; Non ci si aspetta la presenza di allergeni cosmetici e non sono aggiunti intenzionalmente	ALLERGENS	Free from food allergens (Reg. 1169/2011/EU, Annex II); It does not contain gluten; The presence of cosmetic allergens is not expected and they are not intentionally added
GRANULOMETRIA	>= 90,0% passa attraverso 300 micron	PARTICLE SIZE	>= 90,0% through 300 micron
MANIPOLAZIONE	Manipolare in locali provvisti di un buon sistema di aerazione; impedire il contatto delle polveri con sorgenti di accensione, quali fiamme libere, scintille, ecc	HANDLING	Handle in well ventilated room, avoid the contact of powder with fire sources, such as flames and sparks, etc
CONSERVAZIONE	Conservare in contenitori ben chiusi, al riparo da luce, calore e umidità	STORAGE	Store in well closed containers, away from light, heat and moisture
PROPRIETÀ	Uso esterno: condizionante cutaneo	PROPERTIES	External use: skin conditioner
BIBLIOGRAFIA	Il Nuovo Codex Vegetabilis - G. Proserpio; Cosing - European Commission Database	BIBLIOGRAPHY	Il Nuovo Codex Vegetabilis - G. Proserpio; Cosing - European Commission Database

ANNOTAZIONI		NOTES	
NOTE	AVVERTENZA IMPORTANTE Qualora a seguito dell'uso del prodotto insorgano dei disturbi a carico ad esempio della funzione epatica o del sistema nervoso centrale, interrompere l'assunzione e sentire il parere del medico; Esente da OGM (Reg. 1829-1830/2003/CE); esente da rischio BSE/TSE; Non irradiato; Non contiene sostanze classificate C.M.R.; Non contiene nanomateriali; Non testato sugli animali	NOTES	IMPORTANT WARNING If, following the use of the product, disturbances arise in the liver function or central nervous system, for example, stop the intake and seek the advice of the doctor; GMOs-free (Reg. 1829-1830/2003/EC); BSE/TSE-free; Not irradiated; It does not contain C.M.R. substances; It does not contain nanoparticles/nanomaterials; It has not been tested on animals

Le informazioni sopra riportate non vi esonerano dall'obbligo di identificare e controllare il prodotto prima dell'uso. L'adozione dei prodotti e di conseguenza l'uso corretto degli stessi sono sotto la totale responsabilità dell'utilizzatore.

All specifications are as provided by the original manufacturer. They do not imply any exemption from identifying and inspecting the product before its use, the final user being fully responsible for the adoption and the correct usage of the product.

Responsabile Qualità / Head of Quality
Dott.ssa Mariagrazia Roselli

Figure S1. Technical data sheet of the Garcinia cambogia fruit standardized extract.
